# Role of advanced glycation end products in diabetic vascular injury: molecular mechanisms and therapeutic perspectives

**DOI:** 10.1186/s40001-023-01431-w

**Published:** 2023-12-02

**Authors:** Jing Liu, Shuo Pan, Xiqiang Wang, Zhongwei Liu, Yong Zhang

**Affiliations:** 1 Department of Cardiology, Shaanxi Provincial People’s Hospital, 256 Youyi Xi Rd, Xi’an, 710068 China; 2 Affiliated Shaanxi Provincial People’s Hospital, Medical Research Institute, Northwestern Polytechnical University, Xi’an, China

**Keywords:** Advanced glycation end products, Diabetes, Vascular injury, Diabetic cardiovascular complications

## Abstract

**Background:**

In diabetic metabolic disorders, advanced glycation end products (AGEs) contribute significantly to the development of cardiovascular diseases (CVD).

**Aims:**

This comprehensive review aims to elucidate the molecular mechanisms underlying AGE-mediated vascular injury.

**Conclusions:**

We discuss the formation and accumulation of AGEs, their interactions with cellular receptors, and the subsequent activation of signaling pathways leading to oxidative stress, inflammation, endothelial dysfunction, smooth muscle cell proliferation, extracellular matrix remodeling, and impaired angiogenesis. Moreover, we explore potential therapeutic strategies targeting AGEs and related pathways for CVD prevention and treatment in diabetic metabolic disorders. Finally, we address current challenges and future directions in the field, emphasizing the importance of understanding the molecular links between AGEs and vascular injury to improve patient outcomes.

## Introduction

Diabetic metabolic disorders are a heterogeneous group of conditions characterized by chronic hyperglycemia resulting from defects in insulin secretion, action, or both, and are associated with a wide range of complications, including microvascular and macrovascular diseases [1]. Cardiovascular disease (CVD) represents a major cause of morbidity and mortality in patients with diabetes, accounting for approximately 50% of all diabetes-related deaths [2]. Hence, understanding the pathophysiological mechanisms underlying the development and progression of CVD in the context of diabetes is critical for the identification of novel therapeutic targets and strategies to improve patient outcomes.

One of the key factors implicated in the development of diabetes-associated CVD is the accumulation of advanced glycation end products (AGEs), a heterogeneous group of molecules formed by the non-enzymatic glycation of proteins, lipids, and nucleic acids [3]. AGEs have been shown to contribute to the initiation and progression of atherosclerosis, vascular inflammation, and endothelial dysfunction, among other pathological processes, ultimately leading to increased CVD risk in diabetic individuals [4, 5].

**Fig. 1:**
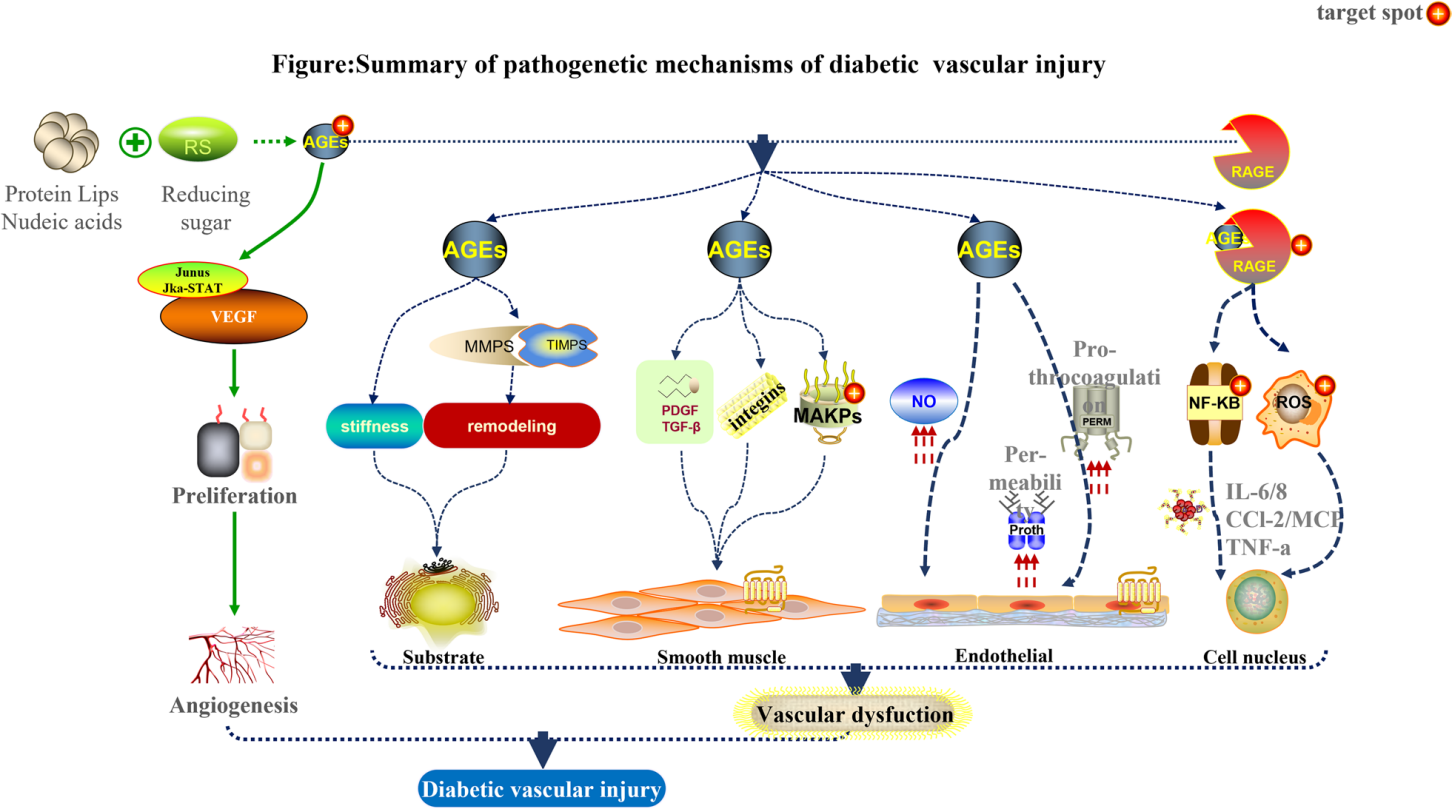
Simplified overview of pathogenetic mechanisms and therapeutic targets by AGEs-induced diabetic vascular injury. *NF-κB* nuclear factor-kappa B, *ROS* reactive oxygen species, *IL-6/8* interleukin-6/8, *CCL-2* chemokine ligand-2, MCL; *TNF-α* tumor necrosis factor, *NO* nitric oxide, *MAPKs* mitogen-activated protein kinases, *PDGF* platelet-derived growth factor, *TGF-β* transforming growth factor-beta, *MMPs* matrix metalloproteinases, *TIMPs* tissue inhibitors of metalloproteinases, *JKA–STAT* Janus kinase–signal transducer and activator of transcription, *VEGF* vascular endothelial growth factor

Despite substantial progress in our understanding of the role of AGEs in the pathogenesis of diabetic CVD, the precise molecular mechanisms linking AGEs to vascular injury remain incompletely understood. Elucidating these mechanisms is essential for the development of targeted therapeutic strategies aimed at preventing or reversing vascular damage in diabetes. In this review, we provide an in-depth overview of the current knowledge on the molecular mechanisms of vascular injury mediated by AGEs (see Fig. 1), focusing on their interactions with key cellular signaling pathways, receptors, and effector molecules implicated in the pathogenesis of diabetic CVD [6, 7].

## Formation and accumulation of AGEs

### Non-enzymatic glycation process

Advanced glycation end products (AGEs) are formed through a non-enzymatic glycation process, wherein reducing sugars react with free amino groups of proteins, lipids, and nucleic acids [8]. This spontaneous reaction occurs under physiological conditions, but its rate is markedly increased in hyperglycemic states, such as diabetes [9]. As a result, diabetic individuals tend to have elevated levels of AGEs, contributing to the development of diabetic complications, including cardiovascular disease [4].

### The Maillard reaction

The formation of AGEs is initiated by the Maillard reaction, a complex cascade of reactions that begins with the reversible condensation of a reducing sugar and a free amino group of a protein, lipid, or nucleic acid, resulting in the formation of a Schiff base [10]. This Schiff base undergoes rearrangement to form a more stable Amadori product. Over time, these early glycation products undergo a series of further reactions, including dehydration, oxidation, and condensation, leading to the formation of irreversible AGEs [11].

### Key AGE precursors and their sources

Various AGE precursors have been identified, including glucose-derived Amadori products, such as glycated hemoglobin (HbA1c), and highly reactive dicarbonyl compounds, such as methylglyoxal, glyoxal, and 3-deoxyglucosone [12]. AGE precursors can be generated endogenously through normal metabolic pathways, including glycolysis, lipid peroxidation, and protein degradation [13]. In addition, exogenous sources of AGE precursors, such as diet, smoking, and environmental pollutants, contribute to the overall AGE burden [14].

### Factors influencing AGE formation

AGE formation is influenced by several factors, including the concentration of reducing sugars and AGE precursors, the presence of transition metal ions, oxidative stress, and the nature of the target proteins or lipids [15]. Furthermore, the rate of AGE accumulation is determined by the balance between their formation, degradation, and clearance. Impaired clearance or increased formation, as observed in diabetes, can lead to the accumulation of AGEs, which can then contribute to diabetic complications [16].

### Classification of AGEs

AGEs are a diverse group of compounds, including fluorescent cross-linked structures, such as pentosidine, and non-fluorescent adducts, such as Nɛ-(carboxymethyl)lysine (CML) and Nɛ-(carboxyethyl)lysine (CEL) [17]. These compounds can exert their deleterious effects by interacting with specific cell surface receptors, such as the receptor for advanced glycation end products (RAGE), or by modifying the structure and function of proteins, lipids, and nucleic acids [18]. The classification of AGEs, precursors and their related diseases are demonstrated in Table 1.

**Table 1:** Classification of advanced glycation end products (AGEs) and their precursors

Type of AGEs	Precursors	Pathways involved	Related diseases/conditions
N^ε-(carboxymethyl)lysine (CML)	Glucose, Glyceraldehyde-3-phosphate	Non-enzymatic glycation; Maillard reaction	CVD, Diabetic nephropathy
N^ε-(carboxyethyl)lysine (CEL)	Glyoxal, Methylglyoxal	Non-enzymatic glycation; Maillard reaction	CVD, Diabetic retinopathy
Pyrraline	3-Deoxyglucosone, Glyoxal	Non-enzymatic glycation; Maillard reaction	CVD, Alzheimer's disease
Pentosidine	Ribose, Glucose	Non-enzymatic glycation; Maillard reaction	CVD, Osteoarthritis
Methylglyoxal-derived hydroimidazolone (MG–H1)	Methylglyoxal	Non-enzymatic glycation; Maillard reaction	CVD, Diabetic neuropathy
Glyoxal-derived hydroimidazolone (G–H1)	Glyoxal	Non-enzymatic glycation; Maillard reaction	CVD, Alzheimer's disease
Argpyrimidine	Methylglyoxal, Arginine	Non-enzymatic glycation; Maillard reaction	CVD, Diabetic nephropathy

## Interaction of AGEs with cellular receptors

### The receptor for AGEs (RAGE)

The receptor for advanced glycation end products (RAGE) is a multi-ligand member of the immunoglobulin superfamily, expressed on various cell types, including endothelial cells, vascular smooth muscle cells, and immune cells [19]. RAGE plays a critical role in mediating the biological effects of AGEs by binding to a range of AGEs and other ligands, such as high mobility group box 1 (HMGB1) and S100 proteins [20]. Engagement of AGEs with RAGE triggers various downstream signaling pathways, contributing to inflammation, oxidative stress, and ultimately, vascular injury in diabetes and other pathological conditions [20].

### Other AGE-binding proteins and their roles

Apart from RAGE, several other AGE-binding proteins have been identified, including galectin-3, CD36, and scavenger receptors, such as SR-A1, SR-B1, and LOX-1 [21, 22]. These receptors are known to contribute to the cellular uptake, degradation, and clearance of AGEs [23]. Interestingly, some of these receptors have been implicated in atherogenesis and the development of other diabetic complications, suggesting that multiple receptor–ligand interactions might be involved in mediating the effects of AGEs in vascular injury [24]. AGEs-related receptors and potential functions are demonstrated in Table 2.

**Table 2:** Cellular receptors involved in AGE recognition and signaling pathways

Receptor	Binding AGEs	Cellular functions	Signaling pathways involved	Related diseases/conditions
RAGE (Receptor for AGEs)	CML, CEL, Pentosidine, MG-H1	Activation of pro-inflammatory responses	NF-κB, MAPK, ERK1/2, JNK, PI3K/Akt, NADPH oxidase	CVD, Diabetic nephropathy
AGER1/OST-48 (Advanced glycation end product-specific receptor 1)	CML, Pyrraline	Suppression of inflammation, AGER/RAGE antagonist	Suppression of RAGE-mediated signaling	CVD, Diabetic retinopathy
AGER2/SR-B1 (Advanced glycation end product-specific receptor 2)	CML, MG-H1	Endocytosis and clearance of AGEs, Regulation of lipid metabolism	PKC, JAK/STAT, MAPK, NF-κB	CVD, Alzheimer's disease
CD36 (Cluster of Differentiation 36)	CEL, Pentosidine	Macrophage foam cell formation, Oxidized LDL uptake	TLR4/TLR6, NF-κB, JNK, p38 MAPK, NADPH oxidase	Atherosclerosis, CVD
LOX-1 (Lectin-like oxidized LDL receptor-1)	CML, Pyrraline	Endothelial dysfunction, Foam cell formation, Apoptosis	NF-κB, MAPK, NADPH oxidase, Caspases, FAK, Src family kinases	CVD, Diabetic retinopathy

### Cellular signaling pathways triggered by AGE–receptor interaction

The interaction of AGEs with cellular receptors, particularly RAGE, activates multiple intracellular signaling cascades, including mitogen-activated protein kinases (MAPKs), such as extracellular signal-regulated kinase (ERK), c-Jun N-terminal kinase (JNK), and p38 MAPK, as well as the nuclear factor kappa B (NF-κB) and Janus kinase–signal transducer and activator of transcription (JAK–STAT) pathways [18]. These signaling pathways, in turn, regulate the expression of pro-inflammatory cytokines, adhesion molecules, growth factors, and other mediators, promoting oxidative stress, inflammation, cell migration and proliferation, apoptosis, and endothelial dysfunction, ultimately leading to vascular injury in diabetic settings [4, 25].

## Molecular mechanisms linking AGEs to vascular injury

### Oxidative stress and inflammation

Advanced glycation end products (AGEs) play a crucial role in the development of vascular injury in diabetes through the induction of oxidative stress and inflammation. AGEs interact with their cellular receptors, such as RAGE, to promote the production of reactive oxygen species (ROS) and the activation of the nuclear factor-kappa B (NF-κB) signaling pathway [7]. This activation leads to the upregulation of pro-inflammatory cytokines, chemokines, and adhesion molecules, contributing to the recruitment of inflammatory cells and perpetuation of inflammation in the vascular wall [5, 26].

### Endothelial dysfunction

AGEs have been shown to impair endothelial function, a key early event in the development of diabetic vascular complications. AGEs can directly inhibit nitric oxide (NO) production and increase endothelial permeability by promoting the degradation of endothelial glycocalyx and disrupting the tight junctions between endothelial cells [27, 28]. Furthermore, AGEs can activate endothelial cells, leading to the expression of pro-thrombotic factors and the initiation of the coagulation cascade, which contributes to thrombus formation and the progression of vascular injury [29].

### Smooth muscle cell proliferation and migration

AGEs can also promote the proliferation and migration of vascular smooth muscle cells (VSMCs), contributing to neointima formation and arterial remodeling in diabetes. AGEs induce the activation of mitogen-activated protein kinases (MAPKs) and the upregulation of growth factors, such as platelet-derived growth factor (PDGF) and transforming growth factor-beta (TGF-β), which stimulate VSMC proliferation and migration [30, 31]. In addition, AGEs can enhance VSMC adhesion to the extracellular matrix by upregulating the expression of integrins and other cell adhesion molecules [32].

### Extracellular matrix remodeling

The accumulation of AGEs in the extracellular matrix (ECM) contributes to vascular stiffness and impaired vascular function in diabetes. AGEs can cross-link with ECM proteins, such as collagen and elastin, leading to alterations in the biomechanical properties of the vascular wall and reduced compliance [33]. Moreover, AGEs can modulate the expression and activity of matrix metalloproteinases (MMPs) and tissue inhibitors of metalloproteinases (TIMPs), which play crucial roles in ECM remodeling and the balance between ECM synthesis and degradation [34, 35].

### Impaired angiogenesis and neovascularization

Impaired angiogenesis and neovascularization are common features of diabetic vascular complications, and AGEs have been implicated in these processes. AGEs can inhibit the proliferation, migration, and tube formation of endothelial progenitor cells and impair the release of pro-angiogenic factors, such as vascular endothelial growth factor (VEGF) [36, 37]. Furthermore, AGEs can activate the Janus kinase–signal transducer and activator of transcription (JAK–STAT) signaling pathway, which inhibits endothelial cell proliferation and contributes to impaired angiogenesis [38].

## Potential therapeutic strategies targeting AGEs in CVD

Growing body of evidence supports the involvement of AGEs in the pathogenesis of cardiovascular disease (CVD) in diabetic patients, suggesting that therapeutic strategies targeting AGEs may offer potential benefits in this population. In this section, we discuss several approaches aimed at attenuating AGE-related vascular injury, including inhibiting AGE formation and accumulation, blocking AGE–receptor interactions, targeting downstream signaling pathways, and utilizing antioxidant and anti-inflammatory therapies. Concluded potential therapeutic strategies targeting AGEs in cardiovascular diseases are demonstrated in Table 3.

**Table 3:** Potential therapeutic strategies targeting AGEs in cardiovascular disease

Therapeutic strategy	Specific targets/compounds	Mechanism of action	Preclinical/clinical studies	Limitations/challenges
Inhibiting AGE formation and accumulation	Aminoguanidine, Pyridoxamine	Blocking the formation of AGEs by trapping reactive carbonyl species	Preclinical, limited clinical trials	Side effects, limited efficacy
	Alagebrium (ALT-711)	Breaks AGE crosslinks, reduces AGE accumulation	Preclinical, Phase II clinical trials	Safety concerns, Incomplete efficacy
Blocking AGE-receptor interactions	Soluble RAGE, Anti-RAGE antibodies	Competitively inhibits binding of AGEs to RAGE, preventing receptor activation	Preclinical, Phase I clinical trials	Limited efficacy, Immunogenicity
	AGER1/OST-48 overexpression	Enhances AGER1-mediated suppression of RAGE signaling	Preclinical	Challenges in gene therapy delivery
Targeting downstream signaling pathways	Inhibition of NF-κB, MAPK, PI3K/Akt	Reduces AGE-induced pro-inflammatory responses and oxidative stress	Preclinical	Potential off-target effects
	Inhibition of NADPH oxidase	Reduces AGE-induced reactive oxygen species (ROS) production	Preclinical	Potential off-target effects
Antioxidant and anti-inflammatory therapies	N-Acetylcysteine, Vitamin E, Curcumin	Scavenges free radicals and reduces inflammation, indirectly targeting AGE pathways	Preclinical, Some clinical studies	Limited efficacy, Need for specificity

### Inhibiting AGE formation and accumulation

One approach to mitigate the effects of AGEs on vascular injury involves inhibiting their formation and accumulation. Various agents, such as aminoguanidine and pyridoxamine, have been shown to inhibit AGE formation by blocking the Maillard reaction or trapping reactive carbonyl species, thereby preventing their interaction with proteins [39, 40]. Clinical trials evaluating the efficacy of these inhibitors in preventing diabetic complications have yielded mixed results, warranting further investigation [41, 42].

### Blocking AGE–receptor interactions

Another strategy focuses on blocking the interaction of AGEs with their cellular receptors, particularly RAGE. Soluble RAGE (sRAGE), a naturally occurring truncated form of the receptor, has been reported to act as a decoy for AGEs, preventing their interaction with cell surface RAGE [42]. Treatment with sRAGE has demonstrated protective effects in preclinical models of CVD [42]. Moreover, small molecule inhibitors targeting the AGE–RAGE axis have shown promise in inhibiting AGE-induced cellular signaling and preventing diabetic complications in experimental models [43].

### Targeting downstream signaling pathways

Since AGE–receptor interactions trigger various intracellular signaling cascades, targeting these downstream pathways represents another potential therapeutic approach. Inhibition of key signaling molecules, such as mitogen-activated protein kinases (MAPKs), nuclear factor-kappa B (NF-κB), and protein kinase C (PKC), has been shown to attenuate AGE-induced cellular responses and prevent the development of diabetic complications in preclinical studies [44–46].

### Antioxidant and anti-inflammatory therapies

Given the role of oxidative stress and inflammation in mediating AGE-induced vascular injury, antioxidant and anti-inflammatory therapies have been explored as potential treatment options. For instance, N-acetylcysteine, a potent antioxidant and precursor to the endogenous antioxidant glutathione, has been demonstrated to mitigate the adverse effects of AGEs on endothelial function [47]. In addition, anti-inflammatory agents, such as statins and angiotensin-converting enzyme inhibitors, have been reported to ameliorate AGE-mediated vascular damage through their pleiotropic effects on inflammation and oxidative stress [48, 49].

## Challenges and perspectives

Although the therapeutic strategies targeting AGEs in CVD have shown promising results in experimental studies, their clinical application is still limited. Several factors contribute to this, including incomplete understanding of AGE-related mechanisms in vascular injury, poor pharmacokinetics of certain anti-AGE compounds, and potential side effects of therapies targeting multiple signaling pathways [50, 51]. Furthermore, many therapeutic agents exhibit limited efficacy in reducing AGE accumulation and alleviating the pathological consequences of AGE–receptor interactions [7].

Despite these challenges, new technologies and strategies are being developed to improve the targeting of AGEs and enhance the efficacy of AGE-based therapies. Nanotechnology-based drug delivery systems have shown potential in improving the bioavailability and stability of anti-AGE agents [52]. In addition, gene editing technologies such as CRISPR/Cas9 can be employed to modulate the expression of key enzymes and receptors involved in AGE formation and signaling, thus offering new opportunities for therapeutic intervention [53]. Furthermore, combination therapies targeting multiple aspects of AGE biology, including AGE formation, receptor interaction, and downstream signaling, may provide synergistic effects and improve overall treatment outcomes [54].

The continued development of novel therapeutic strategies, as well as a deeper understanding of the molecular mechanisms linking AGEs to vascular injury, will be critical for the translation of AGE-based therapies to clinical practice. Large-scale clinical trials are necessary to validate the safety and efficacy of these therapies, as well as to establish their long-term effects on cardiovascular outcomes [55]. Moreover, personalized medicine approaches, including the identification of genetic and environmental factors that influence individual susceptibility to AGE-mediated vascular injury, may facilitate the development of targeted therapies and improve overall patient care [56].

## Conclusion

Our understanding of the molecular mechanisms linking advanced glycation end products (AGEs) to vascular injury has grown significantly over the years. AGEs contribute to the pathogenesis of cardiovascular disease (CVD) in diabetic metabolic disorders through various mechanisms, including oxidative stress, inflammation, endothelial dysfunction, smooth muscle cell proliferation and migration, extracellular matrix remodeling, and impaired angiogenesis and neovascularization. Recognizing the importance of targeting AGEs and their related pathways in preventing and treating CVD in diabetic metabolic disorders is vital, and offers promising therapeutic opportunities. As our understanding of the molecular processes underpinning AGE-mediated vascular injury deepens, so too will our capacity to design targeted therapies that prevent and ameliorate CVD in patients with diabetic metabolic disorders.

## Data Availability

This declaration is not applicable.
